# Influenza A and B Viruses in Fine Aerosols of Exhaled Breath Samples from Patients in Tropical Singapore

**DOI:** 10.3390/v15102033

**Published:** 2023-09-30

**Authors:** Vincent T. K. Chow, Douglas Jie Wen Tay, Mark I. C. Chen, Julian W. Tang, Donald K. Milton, Kwok Wai Tham

**Affiliations:** 1 Infectious Diseases Translational Research Program, Department of Microbiology and Immunology, Yong Loo Lin School of Medicine, National University of Singapore, Singapore 117545, Singapore; douglastay@nus.edu.sg; 2 Research Office, National Centre for Infectious Diseases, Singapore 308442, Singapore; mark_ic_chen@ncid.sg; 3 Department of Respiratory Sciences, University of Leicester, Leicester LE1 7RH, UK; julian.tang@uhl-tr.nhs.uk; 4 Institute for Applied Environmental Health, University of Maryland School of Public Health, College Park, MD 20742, USA; dmilton@umd.edu; 5 Department of the Built Environment, College of Design and Engineering, National University of Singapore, Singapore 117356, Singapore

**Keywords:** influenza A and B, transmission, fine aerosols, coarse aerosols, infectious fine aerosol shedding

## Abstract

Influenza is a highly contagious respiratory illness that commonly causes outbreaks among human communities. Details about the exact nature of the droplets produced by human respiratory activities such as breathing, and their potential to carry and transmit influenza A and B viruses is still not fully understood. The objective of our study was to characterize and quantify influenza viral shedding in exhaled aerosols from natural patient breath, and to determine their viral infectivity among participants in a university cohort in tropical Singapore. Using the Gesundheit-II exhaled breath sampling apparatus, samples of exhaled breath of two aerosol size fractions (“coarse” > 5 µm and “fine” ≤ 5 µm) were collected and analyzed from 31 study participants, i.e., 24 with influenza A (including H1N1 and H3N2 subtypes) and 7 with influenza B (including Victoria and Yamagata lineages). Influenza viral copy number was quantified using reverse transcription-quantitative polymerase chain reaction (RT-qPCR). Infectivity of influenza virus in the fine particle fraction was determined by culturing in Madin–Darby canine kidney cells. Exhaled influenza virus RNA generation rates ranged from 9 to 1.67 × 10^5^ and 10 to 1.24 × 10^4^ influenza virus RNA copies per minute for the fine and coarse aerosol fractions, respectively. Compared to the coarse aerosol fractions, influenza A and B viruses were detected more frequently in the fine aerosol fractions that harbored 12-fold higher viral loads. Culturable virus was recovered from the fine aerosol fractions from 9 of the 31 subjects (29%). These findings constitute additional evidence to reiterate the important role of fine aerosols in influenza transmission and provide a baseline range of influenza virus RNA generation rates.

## 1. Introduction

Influenza is a highly contagious respiratory illness that has resulted in substantial morbidity and mortality worldwide, and it continues to be a major public health concern [1,2,3]. Currently, a major preventive strategy is vaccination [4,5]. While vaccination is an important preventive measure, supplementation with effective non-pharmaceutical interventions can help to control and mitigate the impact of influenza outbreaks [4,6]. The paucity of data on the relative importance of the different modes of influenza virus transmission in humans (contact, droplet, and aerosol) is a critical gap in the knowledge base required to establish and develop evidence-based infection control measures [7,8]. Assessing the risks and potential for aerosol transmission of various infectious agents has been highly topical over the past decade [9]. As a result, the literature in aerosol transmission and infection control has been expanding significantly [3,7,10,11]. However, details about the exact nature of the droplets produced by various human respiratory activities and their potential to carry and transmit such infectious agents are still poorly understood. With the possible emergence of new influenza A subtypes capable of crossing the species barrier to infect humans and the threat of a future influenza pandemic, understanding how and by whom influenza virus is transmitted can help to ensure that the most effective control strategies are implemented [12,13].

Seasonal influenza infections typically occur during the winter periods in temperate regions [14]. However, this seasonality is less distinct in tropical and sub-tropical regions, where infections may occur throughout the year [15,16]. Studies assessing the shedding of influenza virus by infected individuals have been largely based in temperate climates, whereas limited studies have been conducted in tropical and sub-tropical regions. Thus, it is unclear how comparable the estimates of influenza viral shedding are between different climates, and how this could impact the implementation of infection control measures.

Despite numerous studies reporting the detection of influenza viral RNA in the air, few have been able to recover infectious viruses, owing largely to the limitations of the sampling device. Here, using the Gesundheit-II (G-II) exhaled breath sampler [17], we sought to characterize the emission of influenza virus in exhaled breath generated by community-acquired influenza cases in a university cohort in Singapore, a dense city-state situated in the tropics. In addition, we discuss our findings in comparison with SARS-CoV-2 infection, whose aerosol transmission was met with initial controversy [18]. Our findings provide a baseline range of influenza virus generation rates in the tropics and may guide the implementation of evidence-based infection control measures.

## 2. Materials and Methods

### 2.1. Subject Recruitment and Ethics Statement

Volunteers were recruited from November 2013 to January 2015 at the University Health Centre situated in the National University of Singapore after obtaining informed consent. The study was approved by the National Healthcare Group Domain Specific Review Board (reference number 2011/01883). Individuals aged ≥18 years old with at least two influenza-like symptoms within the preceding 72 h were recruited. The list of symptoms for inclusion were fever (≥37.8 °C), cough, sore throat, runny nose (or blocked nose, or stuffy nose), headache, and myalgia. Subjects were asked to complete a short questionnaire that recorded basic demographic data, clinical symptoms, and medical history. A nasopharyngeal swab was collected and analyzed using a QuickNavi–Flu rapid diagnostic test kit (Denka Seiken, Tokyo, Japan) to initially screen for influenza-infected subjects. Subjects who were positive on the influenza rapid test were invited to provide exhaled breath samples. Some of the rapid test-negative subjects were also asked to provide exhaled breath samples to account for possible false negatives. A second nasopharyngeal specimen and a throat swab were collected for analysis using reverse transcription-quantitative polymerase chain reaction (RT-qPCR) and influenza virus culture. Exhaled breath was collected using the Gesundheit-II sampler, which can separate aerosols into fine (≤5 μm) and coarse (>5 μm) fractions, as described previously [17,19]. Exhaled particles were collected for 30 min during tidal breathing, with intermittent recitation of the alphabet at the 5-, 15-, and 25 min marks. The apparatus was disassembled and cleaned with 0.5% hypochlorite solution between subjects to prevent cross-contamination. The study workflow is summarized in Supplementary Figure S1.

### 2.2. Sample Processing

The coarse fraction was collected by impaction on a polytetrafluoroethylene surface, which was removed and placed in a 50 mL conical tube immediately after sampling. In the laboratory, the impactors were swabbed thrice, end-to-end, with a flocked swab moistened with PBS with 0.1% BSA. The swab was then placed in a 15 mL conical tube containing 1 mL of PBS with 0.1% BSA, and vortexed for 30 sec. The fine particle fraction was concentrated with ultrafiltration using Centricon Plus-70 centrifugal filter units (100 kDa; cat. no. UFC710008, Millipore, Burlington, MA, USA) according to the manufacturer’s instructions. The viral concentrate was topped up to 1 mL with PBS with 0.1% BSA, containing 1000 µg/mL of gentamicin (Gibco, New York, NY, USA). Nasopharyngeal and throat swab samples were vortexed for 30 sec, with the subsequent addition of gentamicin (1000 µg/mL). All samples were stored at −80 °C prior to analysis.

### 2.3. Quantification of Influenza Viral Load

RNA was extracted from 200 µL of aliquoted nasopharyngeal swab and exhaled breath samples using the QIAamp MinElute Virus Spin kit (cat. no. 57704, Qiagen, Venlo, The Netherlands) according to the manufacturer’s instructions. The RNA was eluted in 50 µL of nuclease-free water and stored at −80 °C. RT-qPCR was performed using the ABI Prism 7900HT Fast Real-Time PCR System (Applied Biosystems, Foster City, CA, USA) in standard mode. Sequences for the primers and probes were obtained from the Influenza Division, Centers for Disease Control and Prevention, Atlanta, GA, USA (www.cdc.gov/flu/clsis—accessed on 11 June 2012). Duplicate samples were analyzed using influenza A and B primers and probes. Reactions were prepared with the SuperScript III Platinum One-Step Quantitative RT-PCR System kit (cat. no. 11732-020, Invitrogen, Carlsbad, CA, USA). The cycling parameters were 50 °C for 30 min (reverse transcription), 95 °C for 2 min (Taq polymerase activation), and 45 cycles of PCR amplification (each at 95 °C denaturation for 15 s, combined annealing and extension at 55 °C for 30 s). A standard curve was constructed for each assay using RNA extracted from a stock of influenza A/Puerto Rico/8/1934 virus (cat. no. 10-210-500, Advanced Biotechnologies, Eldersburg, MD, USA) with a concentration of 5.3 × 10^11^ virus particles/mL, or a stock of influenza B/Lee/1940 virus (cat. no. 10-220-500, Advanced Biotechnologies) with a concentration of 8.6 × 10^10^ virus particles/mL. Viral RNA copies in the original samples were calculated from the standard curve, accounting for dilution factors. Only subjects with influenza infection confirmed using RT-qPCR (detected in nasopharyngeal swabs) were included in the data analyses.

### 2.4. Influenza Virus Culture

Confluent Madin–Darby canine kidney or MDCK cells (cat. no. CCL-34, American Type Culture Collection, Manassas, VA, USA) in 24-well plates were washed twice with 1× PBS and replaced with 0.1 mL of virus culture medium (OptiMEM I medium supplemented with penicillin–streptomycin, antimycotic, 0.1% BSA, and 2 µg/mL trypsin treated with of L-1-tosylamide-2-phenylethyl chloromethyl ketone (TPCK)). Duplicate wells were inoculated with 0.1 mL of nasopharyngeal, throat, or fine particle fraction samples. The cells were incubated for 1 h at 35 °C with 5% CO_2_ for viral attachment. After this, 1 mL of virus culture medium was added to each well. The cells were then incubated at 35 °C with 5% CO_2_ and examined daily for cytopathic effect or CPE [20]. Samples without CPE were passaged in MDCK cells on day 7.

### 2.5. Statistical Analyses

Data analyses were performed using GraphPad Prism 8.0.2. The Kruskal–Wallis test was used to compare median viral RNA copies of different sample types. Spearman correlation was utilized to examine the correlation between viral RNA copies in nasopharyngeal swabs with the aerosol fractions. Fisher’s exact test was used to compare categorical variables, while Mann–Whitney U test was used to compare continuous variables. All statistical tests were two-sided, and a *p* value of less than 0.05 was considered statistically significant.

## 3. Results

A total of 174 subjects were screened, and 74 subjects provided exhaled breath samples. Of the 74 subjects, 28 were influenza-positive on the rapid test kit, while the remaining 46 subjects who were negative on the rapid test were invited to participate in order to account for possible false negatives (Supplementary Figure S1). In total, 31 subjects were confirmed to have influenza virus infection using RT-qPCR of nasopharyngeal samples, and were included in the analyses (24 influenza A and 7 influenza B), and this included three subjects who were negative on the rapid test but were assessed to be positive with RT-qPCR. Most of the participants were male (64.5%, 20/31), with a median age of 23 years (range: 19 to 54 years). Only 12 (38.7%) of the participants received influenza vaccination in the past 10 years (from the date of recruitment). The characteristics of the 31 participants with confirmed influenza in the study group are summarized in Table 1.

Most of the influenza-positive samples were subjected to next-generation sequencing of the complete viral genomes to determine their subtypes and phylogenetic relationships [20]. Among the influenza A subtypes were 11 H3N2, 6 H1N1, and 1 H1N2, while the influenza B samples included 3 Victoria and 4 Yamagata lineages, and their sequences are available in the GenBank database [20].

**Figure 1:**
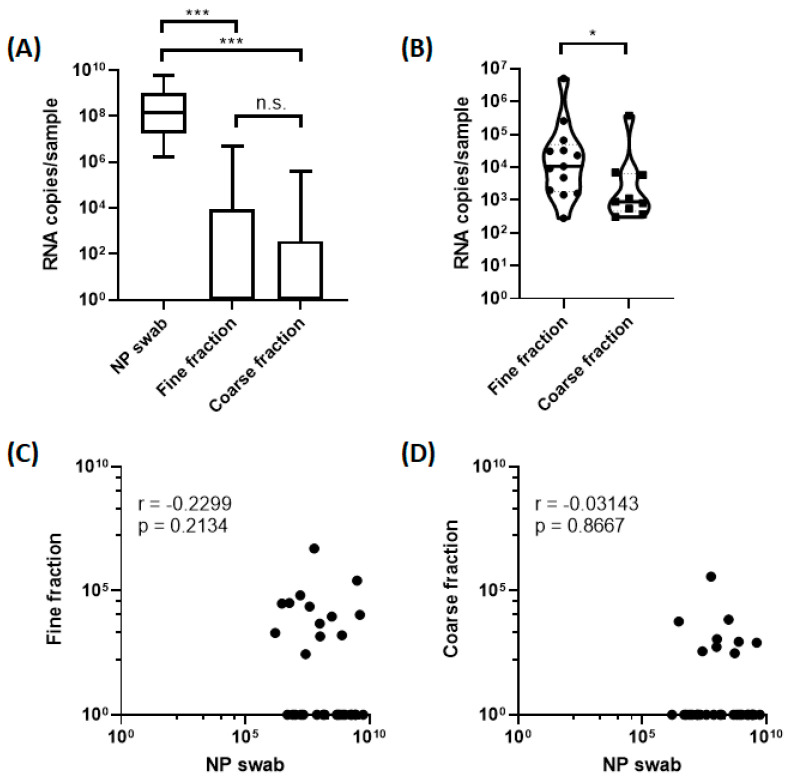
Influenza viral RNA copy numbers in nasopharyngeal swabs and aerosol fractions. (**A**) Comparison of influenza viral RNA copy number in nasopharyngeal samples and fine and coarse aerosol fractions. The Kruskal–Wallis test was performed to compare the median viral RNA copy numbers. (**B**) Comparison of viral RNA copy number in 13 fine versus 9 coarse aerosol fractions of detectable samples. The Mann–Whitney U test was performed to compare the median viral RNA copy numbers. (**C**,**D**) Correlation of viral RNA copy numbers in nasopharyngeal swabs with all (**C**) fine and (**D**) coarse aerosol fractions. Spearman correlation was used to examine the correlation between viral RNA copies in nasopharyngeal swabs with the aerosol fractions. *: *p* < 0.05 (statistically significant). ***: *p* < 0.001 (highly statistically significant).

**Figure 2:**
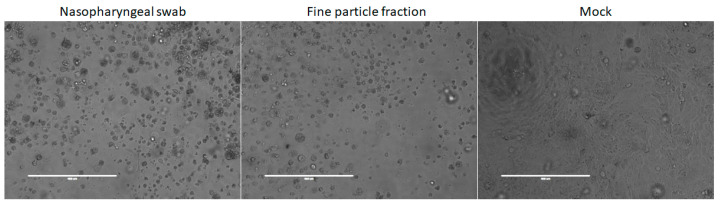
Representative images of influenza virus culture samples indicating infectious influenza virus. Cytopathic effect (CPE) was produced from infected nasopharyngeal swab sample (**left panel**) and fine particle fraction (**center panel**), but not in mock-infected control (**right panel**).

We detected influenza virus RNA in the fine particle fractions collected from 13 (41.9%) of the 31 subjects (9 influenza A and 4 influenza B), and in the coarse particle fractions collected from 9 (29%) of the subjects (7 influenza A and 2 influenza B) as shown in Table 2. Eight (25.8%) subjects had detectable viral RNA copies in both the fine and coarse particle fractions (six influenza A and two influenza B). Fourteen (45%) subjects (ten influenza A and four influenza B) had detectable viral RNA copies in at least one aerosol size fraction. Viral RNA shedding was observed to be the highest in the nasopharyngeal swabs, followed by fine particle fractions and coarse particle fractions (Figure 1A). Excluding samples that had undetectable viral RNA copies, the influenza virus RNA copies in the fine particle fractions ranged from 2.78 × 10^2^ to 5.01 × 10^6^ per 30 min (Table 2 and Figure 1B), corresponding to exhaled breath generation rates ranging from 9 to 1.67 × 10^5^ influenza virus RNA copies per min. Influenza virus RNA copies in the coarse particle fraction ranged from 3 × 10^2^ to 3.73 × 10^5^ per 30 min (Table 2 and Figure 1B), corresponding to exhaled breath generation rates ranging from 10 to 1.24 × 10^4^ influenza virus RNA copies per min. On average, the fine particle fraction (median = 1.07 × 10^4^) exhibited significantly higher (12-fold) influenza virus RNA copies compared to the coarse particle fraction (median = 8.86 × 10^2^; *p* = 0.04). Viral RNA copies in the fine particle fractions were weakly correlated with the viral RNA copies in the nasopharyngeal swabs (*r* = –0.23; Figure 1C). Viral RNA copies in the coarse particle fractions were not correlated with the viral RNA copies in the nasopharyngeal swabs (*r* = –0.03; Figure 1D). We recovered infectious viruses from 28 (90.3%; 21 influenza A and 7 influenza B) nasopharyngeal samples and from 9 (29.0%; 4 influenza A and 5 influenza B) fine particle fraction samples (Table 2 and Figure 2). Age, body temperature, gender, days since symptom onset, symptoms, influenza vaccination in past 10 years, and influenza type were not significantly different between subjects with and without detectable viral RNA copies in the aerosol fractions (Table 3).

To ensure the reproducibility of the above data, the relevant assays were repeated on 16 influenza-positive samples (Table 2) and 6 influenza-negative samples that were randomly selected. The results of these repeated assays were generally consistent and reproducible. A small subset of only 12 out of 31 subjects provided an additional set of samples one day after the first sampling to compare data trends, but these data were not analyzed due to the relatively small sample size.

## 4. Discussion

Although air sampling has demonstrated the presence of influenza virus RNA in the air of healthcare settings, very little is understood about the potential of such infectious agents to be produced and transmitted directly from human respiratory activities [21,22,23]. Various experiments have attempted to capture some of the droplet populations generated by various activities. However, many of these droplets were lost due to settling onto unsampled surfaces, or because only specific droplet parameters were measured. In this study, we utilized a high-efficiency particulate breath collector (G-II) to capture most of the exhaled aerosols during tidal breathing [17].

We detected influenza virus RNA in the fine (42%) and coarse (29%) fractions of subjects with laboratory-confirmed influenza. Viral RNA copy numbers were significantly greater in the fine fraction than in the coarse fraction, consistent with the results obtained from other studies [3,7,19]. Our study measured influenza virus generation rates of 2.8 × 10^2^ to 5.0 × 10^6^ RNA copies per 30 min in the fine particle fraction compared with 3 × 10^2^ to 3.7 × 10^5^ RNA copies per 30 min in the coarse particle fraction. On average, RNA copies in the fine aerosol fraction were 12-fold higher than the coarse aerosol fraction. It was previously suggested that the infectious dose to infect 50% of the population via aerosolization was estimated to be 0.6 to 3 TCID_50_ (assuming 1 TCID_50_ = 650 RNA copies) [24,25]. Assuming a conservative estimate of 3 TCID_50_, this suggests that a 0.7 s exposure (to the highest emitter) to a 3.5 h exposure (to the lowest emitter) to the fine aerosols of an infected individual from our study could result in an infection in 50% of the population. Considering environmental factors (e.g., wind and ultra-violet light) and mechanical factors (e.g., indoor ventilation), it is unlikely that low emitters could be responsible for sustained transmissions. We observed high variability in the amount of influenza viral RNA copies detected in the aerosol fractions, with the highest emitter shedding 2-log_10_ viral RNA copies more than the median viral RNA copy number. This could suggest that certain individuals are more likely to transmit the disease (considered as “super-spreaders”) than others. Thus, an important follow-up study would be to identify the host factors that contribute to high viral shedding and transmission events. In addition, we observed comparable viral shedding with studies performed in other climates that utilized the G-II sampler, suggesting that viral shedding may be more dependent on host factors rather than climatic differences [11,19]. Notwithstanding this, many other factors can play important roles in influenza virus transmissibility, such as gravitational settling rate, wind speed, air temperature, and humidity. These factors can also generate a continuum of aerosol droplet diameter [11].

We observed that there was a lack of correlation between the viral load in the nasopharyngeal and aerosol samples. One reason may be that the droplets produced in the nasopharynx are relatively larger [19]. Another possibility may be that most of the detected droplets originated from the lower respiratory tract where the viral load is not strongly correlated with the nasopharyngeal viral load [26]. In individuals who were nasally inoculated with influenza A, a low proportion of the subjects had detectable viral RNA in the fine aerosol fractions [27]. This suggests that the production of viral aerosols may be dependent on the localization of influenza infection. Moreover, differences in host factors and co-morbidities may be additional variables that influence viral shedding.

To date, there are limited data indicating that fine aerosol particles harbor infectious viruses. In one previous study, infectious influenza virus was detected in 2 of 21 (9.5%) cough aerosol samples in the coarse fraction [3]. Another study recovered viable viruses in 2 of 37 (5.4%), and subsequently in 52 of 134 (39%) of fine particle samples using the G-II breath sampler [10,19]. We recovered infectious virus from the fine aerosol fractions of 9 of 31 (29%) subjects. Coarse particles were not subjected to virus culture because their collection on solid, dry collection media might result in significant loss of infectivity [19]. The finding that infectious influenza virus could be recovered from the fine particle fraction has clinical implications. Firstly, fine aerosol particles are small enough to remain suspended in air, potentially allowing long-range transmissions. Secondly, fine aerosol particles primarily deposit in the lower respiratory tract upon inhalation, which may lead to a higher propensity for severe disease [28,29]. Therefore, strategies to mitigate aerosol transmission are critical considerations to limit the spread of influenza.

The emergence of SARS-CoV-2 has rekindled the debate on the aerosol route of transmission for respiratory viruses. While initially thought to be spread via close contact or fomites, numerous studies have provided evidence of the presence of SARS-CoV-2 viral RNA in the air and of the recovery of infectious virus from air samples [30,31,32,33,34]. In comparison with SARS-CoV-2 infection, we observed higher viral RNA emissions for influenza-infected subjects. In addition, there has been limited success in culturing viable SARS-CoV-2 from exhaled breath samples compared to culturing viable influenza virus using the G-II sampler. This suggests that influenza may be spread more predominantly via the aerosol route compared to SARS-CoV-2, evident by its sharp decline with the implementation of mandatory masking during the COVID-19 pandemic [35]. Despite vaccination, individuals infected with SARS-CoV-2 or influenza virus are still able to emit viral aerosols, which may contribute to continued transmissibility. Therefore, vaccination remains an important preventive measure against contracting the disease, while non-pharmaceutical interventions to mitigate viral aerosols are necessary complementary measures.

Our study has certain limitations. Firstly, our study had a relatively small sample size and limited age range of subjects. Larger studies of exhaled breath particle concentrations are necessary to better characterize the particle generation rates in the population and how these vary between age groups and co-morbidities. Secondly, we did not collect and analyze samples over multiple days for all influenza-positive subjects, which may have revealed any trends between viral shedding and day of illness. Thirdly, we did not detect influenza virus RNA in 55% (17/31) of aerosol samples of the subjects. This may be attributed to the relatively shorter collection time and lack of prolonged respiratory activities such as talking and singing, which could generate more aerosolized particles [36]. In a similar study on patients infected with SARS-CoV-2, we detected higher viral emissions during talking and singing compared to tidal breathing [32,33]. Thus, given that viral shedding during tidal breathing for influenza subjects were orders of magnitude greater than SARS-CoV-2 subjects, higher viral emissions may be expected if influenza subjects were tasked to talk and sing.

In conclusion, this study has shown that aerosolized influenza virus is detected mainly in the fine aerosol fraction from natural patient breath and exhibits infectivity in nearly one-third of subjects. We provide a baseline range of influenza virus RNA generation rates during tidal breathing from an infected study population in the tropics. These findings reiterate the airborne route of influenza transmission and highlight the importance of non-pharmaceutical interventions to reduce exposure to fine aerosols, such as masking and air ventilation and filtration.

## Figures and Tables

**Table 1:** Characteristics of the 31 participants in the study group with influenza confirmed using RT-qPCR.

	Total Subjects (*n* = 31)
**Parameter**	
**Age (years)** Median Range	23 19–54
**Temperature (°C)**	
Median Range	38.2 36.4–39.8
**Male (*n* (%))**	20 (64.5%)
**Onset of symptoms since (*n* (%))** 1 day ago 2 days ago 3 days ago	18 (58.1%) 11 (35.5%) 2 (6.5%)
**Symptoms (*n* (%))** Runny nose Sore throat Headache Sweats or chills Cough	27 (87.1%) 26 (83.9%) 27 (87.1%) 28 (90.3%) 27 (87.1%)
**Influenza vaccination in past 10 years (*n* (%))**	12 (38.7%)
Infected with influenza type A (*n* (%)) *	24 (77.4%)
Infected with influenza type B (*n* (%)) *	7 (22.6%)
**Virus culture positives (*n* (%))** Nasopharyngeal sample Fine aerosol fraction	28 (90.3%) 9 (29.0%)

* Determined using RT-qPCR and/or nucleotide sequencing.

**Table 2:** Influenza viral RNA copies (per 30 min) and virus culture positivity in nasopharyngeal (NP) swab and fine and coarse aerosol fractions.

Sample ID	Influenza Type ^a^	NP Swab ^b^	Fine Fraction ^c^	Coarse Fraction ^c^	Virus Culture Positive
G2-5.1 * (R)	A (H3N2)	2.95 × 10^6^	3.05 × 10^4^	5.78 × 10^3^	NP
G2-6.1	A (H3N2)	1.03 × 10^7^	-	-	NP and F
G2-7.1 (R)	B (Victoria)	5.95 × 10^7^	5.01 × 10^6^	3.73 × 10^5^	NP and F
G2-8.1 (R)	A (H3N2)	4.92 × 10^6^	-	-	NP
G2-9.1 (R)	A (H3N2)	9.94 × 10^7^	4.76 × 10^3^	5.47 × 10^2^	NP
G2-10.1	A (H3N2)	1.36 × 10^8^	-	-	NP and F
G2-14.1 * (R)	B (Victoria)	1.62 × 10^7^	6.58 × 10^4^	-	NP and F
G2-15.1 *	B (Victoria)	1.71 × 10^9^	-	-	NP and F
G2-19.1	A (H1N1)	1.93 × 10^9^	-	-	NP
G2-20.1 (R)	A (H1N1)	5.92 × 10^6^	3.21 × 10^4^	-	NP
G2-22.1	A (H1N1)	5.52 × 10^9^	-	-	NP
G2-23.1 (R)	A (H1N1)	7.96 × 10^6^	-	-	NP
G2-24.1 *	B (Yamagata)	8.27 × 10^8^	-	-	NP
G2-25.1*	A (H1N1)	7.63 × 10^7^	-	-	NP
G2-26.1 * (R)	A (H3N2)	5.35 × 10^8^	-	3.03 × 10^2^	NP
G2-27.1 *	A (H1N2)	2.75 × 10^9^	-	-	NP
G2-29.1 *	A (H3N2)	4.76 × 10^8^	-	-	NP
G2-31.1	A (H3N2)	2.14 × 10^7^	-	-	NP
G2-34.1 * (R)	B (Yamagata)	3.85 × 10^7^	2.28 × 10^4^	-	NP
G2-36.1	B (Yamagata)	1.56 × 10^8^	-	-	NP and F
G2-37.1 *	A	1.03 × 10^9^	-	-	NP and F
G2-41.1 *	A	2.67 × 10^9^	-	-	NP and F
G2-42.1	A	6.46 × 10^8^	-	-	NP
G2-43.1 (R)	B (Yamagata)	4.05 × 10^9^	1.07 × 10^4^	8.02 × 10^2^	NP and F
G2-44.1 * (R)	A (H1N1)	3.15 × 10^9^	2.56 × 10^5^	-	NP
G2-49.1 (R)	A	2.65 × 10^7^	2.78 × 10^2^	3.62 × 10^2^	-
G2-51.1 (R)	A (H3N2)	3.04 × 10^8^	9.08 × 10^3^	6.85 × 10^3^	NP
G2-52.1 (R)	A (H3N2)	1.70 × 10^7^	-	-	-
G2-53.1 (R)	A	1.02 × 10^8^	1.44 × 10^3^	1.13 × 10^3^	-
G2-63.1 (R)	A (H3N2)	7.66 × 10^8^	1.60 × 10^3^	8.86 × 10^2^	NP
G2-72.1	A	1.58 × 10^6^	1.99 × 10^3^	-	NP

*: Vaccinated in last 10 years from date of recruitment; NP: nasopharyngeal; F: fine aerosol fraction; -: influenza not detected using RT-qPCR or viral culture; and (R): assays were repeated with consistent results. ^a^: influenza subtyping and genome sequencing with next-generation sequencing, as previously reported [20]. ^b^: viral RNA copies in original nasopharyngeal swab sample. ^c^: viral RNA copies emitted in aerosols within 30 min of sampling.

**Table 3:** Comparison of variables between subjects with and without detectable influenza viral RNA in respiratory aerosols.

Variable	Detected (*n* = 14) ^a^	Not Detected (*n* = 17) ^b^	*p* Value
Age	23 (19–28 years)	23 (19–54 years)	0.42
Temperature	38.1 (36.4–39.7 °C)	38.4 (37.3–39.8 °C)	0.15
Male	11 (78.6%)	9 (52.9%)	0.26
Days since symptom onset	1 (1–3 days)	1 (1–3 days)	>0.99
Symptoms			
Runny nose	13 (92.9%)	14 (82.4%)	0.61
Sore throat	12 (85.7%)	14 (82.4%)	>0.99
Headache	12 (85.7%)	15 (88.2%)	>0.99
Sweats or chills	12 (85.7%)	16 (94.1%)	0.58
Cough	13 (92.9%)	14 (82.4%)	0.61
Influenza vaccination in past 10 years	5 (35.7%)	7 (41.2%)	>0.99
Infected with influenza type A	10 (71.4%)	14 (82.4%)	0.41

Values are stated as number (percentage) for categorical variables and median (range) for continuous variables. Categorical variables were compared using Fisher’s exact test, and continuous variables were compared using Mann–Whitney U test. ^a^: Viral RNA detected in fine and/or coarse fraction. ^b^: Viral RNA not detected in both aerosol fractions.

## Data Availability

The data presented in this study are available on reasonable request from the corresponding author.

## Supplementary Materials

The following supporting information can be downloaded at: https://www.mdpi.com/article/10.3390/v15102033/s1, Supplementary Figure S1: Study workflow of subject recruitment, sampling, and laboratory assays.
