# Supplementary material for: Influenza A and B Viruses in Fine Aerosols of Exhaled Breath Samples from Patients in Tropical Singapore
Source: Viruses. 2023 Sep 30;15(10):2033. doi: 10.3390/v15102033 (PMC10612062; doi:10.3390/v15102033)
Supplement: Supplementary file 1 [file viruses-15-02033-s001.zip › viruses-2611327-supplementary.pdf]

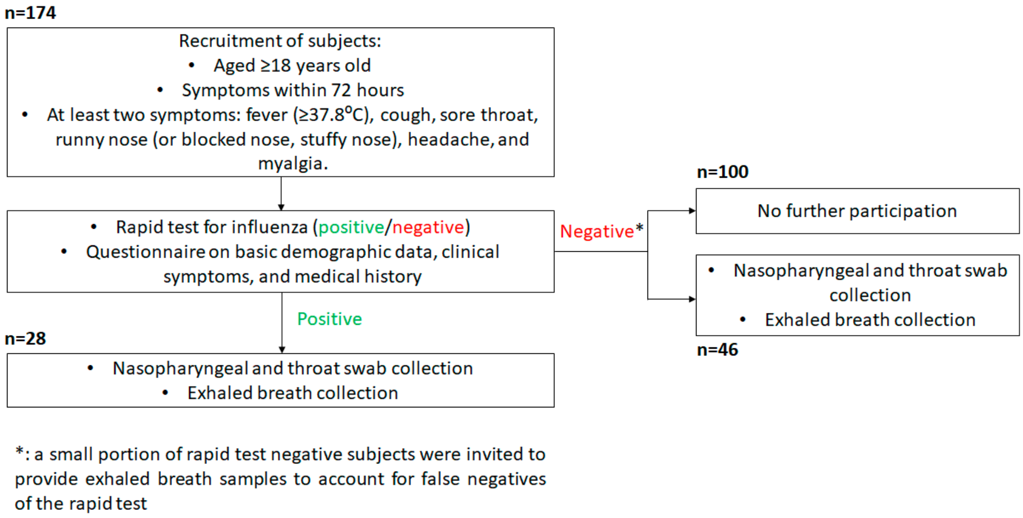

Supplementary Figure S1. Study workflow of subject recruitment, sampling and laboratory assays.
